# Bayesian model selection for the Drosophila gap gene network

**DOI:** 10.1186/s12859-019-2888-0

**Published:** 2019-06-13

**Authors:** Asif Zubair, I. Gary Rosen, Sergey V. Nuzhdin, Paul Marjoram

**Affiliations:** 1 Molecular and Computational Biology, USC, 1050 Childs Way, Los Angeles, CA 90089-2532 US; 2 Department of Mathematics, USC, 3620 S. Vermont Ave., Los Angeles, CA 90089-2532 US

**Keywords:** Gap genes, Reaction-diffusion equations, Bayesian model selection, Parallel tempering, Bayes factor

## Abstract

**Background:**

The gap gene system controls the early cascade of the segmentation pathway in *Drosophila melanogaster* as well as other insects. Owing to its tractability and key role in embryo patterning, this system has been the focus for both computational modelers and experimentalists. The gap gene expression dynamics can be considered strictly as a one-dimensional process and modeled as a system of reaction-diffusion equations. While substantial progress has been made in modeling this phenomenon, there still remains a deficit of approaches to evaluate competing hypotheses. Most of the model development has happened in isolation and there has been little attempt to compare candidate models.

**Results:**

The Bayesian framework offers a means of doing formal model evaluation. Here, we demonstrate how this framework can be used to compare different models of gene expression. We focus on the Papatsenko-Levine formalism, which exploits a fractional occupancy based approach to incorporate activation of the gap genes by the maternal genes and cross-regulation by the gap genes themselves. The Bayesian approach provides insight about relationship between system parameters. In the regulatory pathway of segmentation, the parameters for number of binding sites and binding affinity have a negative correlation. The model selection analysis supports a stronger binding affinity for Bicoid compared to other regulatory edges, as shown by a larger posterior mean. The procedure doesn’t show support for activation of Kruppel by Bicoid.

**Conclusions:**

We provide an efficient solver for the general representation of the Papatsenko-Levine model. We also demonstrate the utility of Bayes factor for evaluating candidate models for spatial pattering models. In addition, by using the parallel tempering sampler, the convergence of Markov chains can be remarkably improved and robust estimates of Bayes factors obtained.

**Electronic supplementary material:**

The online version of this article (10.1186/s12859-019-2888-0) contains supplementary material, which is available to authorized users.

## Background

In this paper, we explore models for the developmental process of segmentation in *Drosophila*, providing an efficient model solver. We use the Bayesian framework for inference and model selection. The process by which multicellular organisms develop from a single fertilized cell has been the focus of much attention. It was postulated that organisms are patterned by gradients of certain form-producing substances. Boveri [1] and Horstadius [2] used this idea to explain the patterning of the sea urchin embryo. The idea was given further impetus by the discovery of the Spemann organizer [3] which suggested that morphogenesis is the result of signals released from localized group of cells. In 1952, Turing, working on the problem of spatial patterning, coined the term morphogen to describe ‘form-producers’. He used mathematical models to show that chemical substances could self-organize into patterns starting from homogeneous distributions [4]. However, a definitive example of a morphogen was only provided in 1987 by the discovery of Bicoid function in the *Drosophila* embryo [5, 6] and subsequent visualization of its gradient [7, 8]. Not surprisingly, patterning in the *Drosophila* embryo has been the focus of both developmental and systems biologists.

The formation of several broad gap gene [9] expression patterns within the first two hours of development characterizes early *Drosophila* embryogenesis. Taken together, the gap genes constitute one of the four regulatory layers in the cascade of segmentation pathway in *Drosophila* embryo. Expression of gap genes is regulated by maternal genes [10] and they also participate in mutual repression [11]. Thus, activation by maternal gradients, combined with spatially specific gap-gap cross repression helps to establish, sharpen and maintain the broad overlapping domains of the gap gene expression along the Anterior-Posterior (A-P) axis. The gap gene network is one of the few examples of a developmental gene network which has been studied extensively using data-driven mathematical models [12–14] in order to reconstruct the regulatory structure of the gap gene network. However, there continues to be active discussion [15, 16] on how maternal gradients and mutual gap gene repression contribute to the formation of gap stripes.

Mathematical representation of the gap gene network through quantitative dynamical systems has helped investigate regulatory structure of this network along with specific properties of this representation such as the strength of interaction, cooperativity of regulators, etc. However, there is a deficit of a rigorous framework within which putative representations can be compared and allows one to conduct formal statistics of relative fit. In a seminal paper, Jaeger et al. [12] used a dynamical model where a genetic inter-connectivity matrix described the regulatory parameters. Based on measures of model fit, they argued that dual regulatory action of Hunchback on Kruppel is not essential for to explain gap gene domain formation. While this may be valid, they do not provide a relative goodness of fit of the model against a representation that assumes dual-regulation. Perkins et al. [17] did an extensive study of gap gene regulatory relationships and compared proposed networks in literature. However, their study does not provide a measure of statistical significance for model comparison. Essentially, the question we want to ask is how to chose between competing hypothesis for the network structure in a statistically rigorous manner ? In addition, real data is often contaminated with measurement noise and we need methods that can help us deal with this uncertainty.

Addressing the latter point, one way to handle error associated with experimental observations is to model it as Gaussian noise. If we know or are willing to assume a model for the error variance, then an estimate of the parameters can be sought by maximizing the likelihood in a least squares sense. This is the maximum likelihood estimate (MLE) [18] of the parameters. However, this point estimate suffers from being unrepresentative and is often intractable, especially if the likelihood is multimodal.

An alternative approach is the Bayesian framework which allows one to not only account for experimental error by propagating it to the model parameters but also a way to integrate our prior beliefs on the distribution of model parameters. In this manner, a posterior distribution of the model parameters is obtained which encapsulates our belief in the parameter values given uncertainty in measurement. Indeterminacy of model parameters and correlations between indeterminate parameters are incorporated into the marginal likelihood (evidence). Direct computations of integrals involved in Bayesian methods are difficult and so researchers tend to use Markov chain Monte Carlo (MCMC) methods like Gibbs sampling or Metropolis-Hastings algorithm [19]. Bayesian approaches have enjoyed great success in genetics [20] and we and others [21] expect that they will provide more satisfactory solutions to inference problems in computational systems biology.

In addition, the Bayesian approach allows us to assess which of the competing models is better supported by the data by comparing the ratios of marginal likelihood of the models. The process of comparing models is more formally known as model selection and the ratio of marginal likelihoods is also called the Bayes factor [22]. It follows, that in order to use Bayes factors, one needs to estimate the marginal likelihood of a model. However, this task becomes increasingly intractable with growing model dimensionality and a conventional Metropolis-Hastings sampling approach generally leads to poor mixing properties and unreliable conclusions. To overcome this difficulty, we use the parallel tempering Markov chain Monte Carlo (PT-MCMC) sampling technique [23]. Briefly, this method runs parallel chains at different temperatures (or degree of smoothness of likelihood surface) and allows exchanges between the chains based on the Hastings ratio. The end result is a chain that mixes well and also doesn’t get stuck in local optima. Another benefit of this approach is that it allows one to use path integration to compute the thermodynamic estimator [24] of the marginal likelihood. This estimator has been shown to be reliable when working with Bayes factors [25] in the context of differential equations.

We currently focus on the Papatsenko-Levine formalism [26], which exploits a fractional occupancy based approach to incorporate activation of the gap genes by the maternal genes and cross-regulation by the gap genes themselves. An advantage of this formalism is that it incorporates non-linear effects between regulatory interactions and is closer to a mechanistic view of how regulation in this system occurs [27]. While in their paper, Papatsenko & Levine assumed that network structure is known a priori, our approach allows one to choose from competing network topologies reported in the literature and to vary strength of interactions between gap genes. It is worth mentioning here that although we consider models of increasing complexity, Bayes factors allows model comparison without concerns of over-fitting, that is, they allow one to implicitly control for model dimensionality [28].

## Methods

### Expression data

**Fig. 1:**
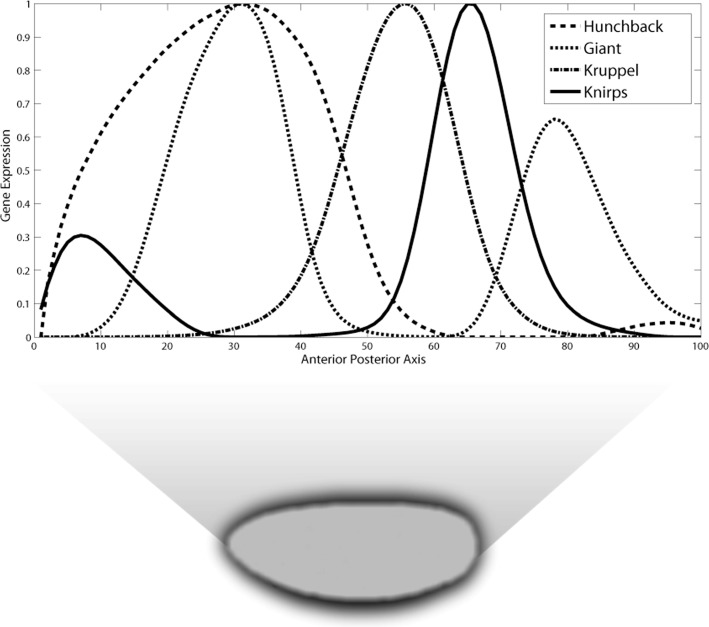
Expression data. Gap gene expression values at cleavage cycle 14.4 along the anterior-posterior axis of developing embryo are used to fit the model

We use published data by Papatsenko & Levine [26]. This data was obtained from the FlyEx database [29]. The data comprise of expression values on a line along the Anterior-Posterior axis of the embryo and subsampled to 100 spatial points separated by approximately 5*μm*. Maternal Bicoid (Bcd) and Hunchback (Hb) expression data corresponding to cleavage cycle 14.1 were used as input to the model. The output data is gap gene zygotic expression at cleavage cycle 14.4 for Hunchback, Kruppel (Kr), Knirps (Kni) and Giant (Gt) (Fig. 1). Tailless (Tll) expression data corresponding to cleavage cycle 14.4 was also used as input.

### Model solution

Time-varying systems can be modeled with ordinary differential equations (ODEs) which have efficient solvers available (for example, [30]). However, in pattern formation gene expression varies both in time and space and partial differential equations (PDEs) are the suitable method for characterizing this process. Closed form solutions for PDEs exist only in the most simplest of cases and numerical solutions need to be employed. Packaged solvers for PDEs do exist [31] and some like deal.II [32] have been used in systems biology applications [33–35]. However, due to the overhead of generalizability and computational tractability in structuring models, we wrote our own solver.

We first elaborate the PDE formalism, due to Papatsenko & Levine, used for describing gap gene expression:

$$\begin{array}{*{20}l} \frac{\partial }{\partial t}u_{i}(x,t) &= \alpha P_{i}^{A}\left(1 - P_{i}^{B}\right) - \beta u_{i}(x,t) + D \frac{\partial^{2} u_{i}(x,t)}{\partial x^{2}}, \\ i &= Hb, Kr, Kni, Gt, \\ u^{\prime}(0,t) &= u^{\prime}(L,t) = 0, u^{\prime} = \frac{\partial u}{\partial x}, \\ 0 &< x < L, 0 < t < T. \end{array} $$

Here, *u*_*i*_(*x*,*t*) represents the expression of gap gene, *i*, at time *t* and position *x* with Neumann boundary conditions, i.e., we assume that flux at the boundaries is zero. *α* represents the production rate, *β* is the linear decay rate and *D* is the diffusion constant. *L* denotes the length of the embryo and *T* corresponds to cleavage cycle 14.4 which marks the start of gastrulation. *P*^*A*^ and *P*^*B*^ are respectively combined activation and repression effects of regulators for each gap gene. These regulatory effects are a function of the gap gene expression and its binding affinity (*K*), cooperativity rate (*C*_*o*_) and the number of binding sites (*N*_*s*_). (Details in the Additional file 1 text.)

We reformulate the system in weak or variational form [36] and then rely on the theory of linear semigroups of operators [37]. We point the interested reader to the supplementary material in Additional file 1 for a full derivation of the solution. The observed data is assumed to have some noise *ε*, which we take to be identically normally distributed, *ε*∼*N*(0,*σ*^2^*I*), (where *I* is the identity matrix). If the observed data is *Y* and *U* is the solution to the system of PDEs, we have:

$$\begin{array}{*{20}l} Y_{i} &= U_{i}(x,T) + \epsilon, \\ i &= Hb, Kr, Kni, Gt, \\ 0 &< x < L. \end{array} $$

### Parameter estimation

Following the above formulation, we can define the likelihood function, *L*(*θ*,*Y*), which gives the conditional probability of the data, *Y*, given the parameter, *θ*. Here we have the dropped the subscript *i* for gap genes for the sake of convenience. Given the assumed error model, the likelihood can be written down explicitly as

$$L(\theta, Y) = p(Y | \theta) = \prod_{j=1}^{N} \frac{1}{\sqrt{2 \pi \sigma^{2}}} \textup{exp}\left(-\frac{1}{2 \sigma^{2}}(y_{j} - u_{j})^{2}\right). $$

We note that we apply the error model for specific domains over the embryo length (e.l.). Specifically, the domains used for the gap genes are 30-70% e.l. for Hb, 40-90% e.l. for Kni, 20-80% e.l. for Kr and 10-90% e.l. for Gt. The posterior incorporates both how well the parameters support the data and also our existing knowledge of them. This can be expressed more mathematically using Bayes’ theorem [38]:

$$p(\theta | Y) = \frac{L(\theta, Y) \pi(\theta)}{p(Y)} $$

where

- *p*(*θ*|*Y*) is the posterior density of the parameters
- *L*(*θ*;*Y*) is the likelihood of the data as elaborated above
- *π*(*θ*) is the prior belief of the parameter
- *p*(*Y*) is the marginal likelihood

At first glance, it would appear straightforward to use Bayes’ theorem to compute the posterior density of the parameters. However, the marginal likelihood term in the denominator is often hard to evaluate numerically and mostly intractable as it involves an integration of the likelihood over the whole parameter space:

$$p(Y) = \int_{\Theta} L(\theta, Y) \pi(\theta) d\theta. $$

Instead, we rely on the Markov chain Monte Carlo [39] method used for high-dimensional sampling. The idea behind these methods is to draw samples from the stationary distribution of a Markov chain. When set up correctly, this distribution produces samples from the posterior distribution. The marginal likelihood itself, however, is relevant for model selection and we will return to its estimation in the “Model selection” section.

#### Metropolis-Hastings sampling

The Metropolis-Hastings algorithm [19] provides a procedure to draw samples from the target distribution based on a proposal density. When the appropriate target density is defined, this amounts to generating samples from the posterior distribution of the dynamic model of interest. The MH algorithm achieves this by suggesting moves based on a proposal distribution, *q*(*θ*_*i*+1_|*θ*_*i*_), for the Markov chain which proposes a new value for *θ*_*i*+1_ conditional on the current value of *θ*_*i*_. These moves are accepted based on the Hastings ratio:

$$\begin{aligned} a_{hr} &= min\left \{ 1, \frac{p(\theta_{i+1}| Y) q(\theta_{i}| \theta_{i+1})}{p(\theta_{i}| Y) q(\theta_{i+1}| \theta_{i})} \right \}\\ &= min\left \{ 1, \frac{L(\theta_{i+1}, Y) \pi(\theta_{i+1})q(\theta_{i}| \theta_{i+1})}{L(\theta_{i}, Y) \pi(\theta_{i})q(\theta_{i+1}| \theta_{i})} \right \}. \end{aligned} $$

The terms are as defined previously and we note that the marginal likelihood term has conveniently canceled out in denominator. The proposal *q*(·|·) is usually taken to be a Gaussian, however, we note that in our case, the number of sites parameter, *N*_*s*_, is discrete. Accordingly, we define the proposal density as a mixed density. With probability, *p*<1/10, we perturb *N*_*s*_ by either increasing or decreasing it by 1 with equal probability, while keeping the rest of the parameters unchanged. Else, we perturb each of the other parameters based on a Gaussian centered at the current value of the parameters, *θ*_*i*_ and with variance 0.1*I*, where I is the identity matrix. We use bounded uniform prior on all the parameters.

#### Parallel tempered MCMC sampling

In principle, given a large number of samples, the Metropolis-Hastings sampler should be able to cover the whole parameter space. However, in high dimensions, the number is samples required increases rapidly and there is always the chance of the chain getting stuck in local optima. To get around these issues, it has been proposed to use multiple interacting MCMC chains [23]. One such approach is of parallel tempering where parallel MCMC chains are run at different ’temperatures’. The range of temperatures that are used is referred to as the temperature ladder. The likelihood for a chain at temperature *t* is now given by:

$$L_{t}(\theta, Y) = p_{t}(Y| \theta) = p(Y| \theta)^{t}. $$

Since the likelihood function is smoother for higher temperatures, chains at higher temperature can sample the parameter space more freely. The chains are updated using a Metropolis Hastings update step and chains at neighbouring temperatures are exchanged using an acceptance ratio. For implementation purposes, we follow the approach in [40] with a slight modification. Algorithmically:

1. Initial start positions are assigned to each chain, *Θ*=(*θ*_1_,…,*θ*_*N*_)
2. Associate each chain with a temperature based on a temperature ladder, (*Θ*,*t*)=(*θ*_1_,*t*_1_,…*θ*_*N*_,*t*_*N*_)
3. Repeat till convergence of all chains
  - Apply local Metropolis-Hastings update step to each chain
  - Pick two neighboring chains at different temperature. Assume states *θ*_*i*_ and *θ*_*j*_ for N pairs (*i*,*j*) with *i* sampled uniformly in (1,…,*N*) and *j*=*i*±1 with probability *p*_*e*_(*θ*_*i*_,*θ*_*j*_) where *p*_*e*_(*θ*_*i*_,*θ*_*i*+1_)=*p*_*e*_(*θ*_*i*_,*θ*_*i*−1_)=0.5 and *p*_*e*_(*θ*_1_,*θ*_2_)=*p*_*e*_(*θ*_*N*_,*θ*_*N*−1_)=1
  - Exchange the state of the chains based on acceptance ratio
4. Use chain with lowest temperature for estimating posterior density

The exchange step is accepted with probability *min*(1,*a*_*e*_) according to the Metropolis-Hastings rule:

$$\begin{aligned} a_{e} &= \frac{p(\Theta^{\prime}|Y)Q(\Theta | \Theta^{\prime})}{p(\Theta |Y)Q(\Theta^{\prime}| \Theta)}\\ &= \frac{[L(\theta_{j},Y)^{t_{i}}*L(\theta_{i},Y)^{t_{j}}]}{[L(\theta_{i},Y)^{t_{i}}*L(\theta_{j},Y)^{t_{j}}]}* \frac{Q(\Theta | \Theta^{\prime})}{Q(\Theta^{\prime}| \Theta)} \end{aligned} $$

where *Q*(·|·) denotes the probability of transition from a set of chains to a set with a neighboring pair of chains exchanged. We select direct neighbors in the temperature ladder for the exchange step to increase the likelihood for the exchange to be accepted.

While the chain at the lowest temperature can be used for parameter inference, all the chains together can be used to estimate the marginal likelihood [25] and in turn calculate Bayes factors for Bayesian model comparison for model ranking. It is this aspect that we turn to next.

### Model selection

In the context of Bayesian inference, Bayes factors can be employed to do model selection. They allow us to compute the posterior probabilities of two models, given the prior probability of each model. Assuming again that the data is *Y*, and we want to compare between two models, *M*_1_ and *M*_2_, then the posterior odds are given by:

$$\frac{p(M_{1}|Y)}{p(M_{2}|Y)} = \left (\frac{p(Y|M_{1})}{p(Y|M_{2})} \right) \frac{p(M_{1})}{p(M_{2})}. $$

The quantity in brackets is the ratio of the marginal likelihoods of the two models and is termed the Bayes factors. When we have no prior preference of one model over the other, we assume *p*(*M*_1_)=*p*(*M*_2_) and then the ratio of likelihoods is exactly equal to the Bayes factor. In essence, then, the problem of model selection boils down to the problem of estimating the marginal likelihood.

Various methods to estimate the marginal likelihood have been proposed [41, 42]. In the simplest construction, given samples from the prior *θ*_1_,*θ*_2_,...,*θ*_*n*_, one could compute the Monte Carlo estimate

$$\hat{p}(Y) = \frac{1}{n}\sum_{i=1}^{n}p(Y|\theta_{i}). $$

However, in practice this is a poor estimator unless working with very large sample sizes. Similarly, the importance sampling based the posterior harmonic mean estimator has been shown [42, 43] to be a very poor estimator.

Instead, we could exploit the tempered distributions that we have generated using the PT-MCMC sampler. This approach has been referred to as path sampling [24, 43]. If we assume that the marginal likelihood of chain at temperature *t* is represented as *z*_*t*_, then:

$$z_{t} = z(t) = \int_{\Theta} p(Y|\theta)^{t}\pi(\theta) d\theta. $$

By differentiating the logarithm of *z*,

$$\begin{aligned} \frac{d}{dt}\text{log}z_{t} &= \int_{\Theta}\text{log}(p(Y|\theta)) \cdot \frac{p(Y|\theta)^{t} \pi(\theta)}{z_{t}} d\theta\\ &= E_{t}[\text{log}(p(Y|\theta))] \end{aligned} $$

and then we can integrate both sides with respect to *t* to obtain:

$$\text{log}(p(Y)) = \int_{0}^{1}E_{t}[\text{log}(p(Y|\theta))]dt $$

as described in [41]. Thus, if we choose a temperature ladder (0=*t*_0_<*t*_1_<*t*_2_<...<*t*_*N*−1_<*t*_*N*_=1), then we can use a numerical approximation to compute the above integral. Namely,

$$\begin{aligned} \text{log}(p(Y)) &= \sum_{i=1}^{N-1}0.5(t_{i+1} - t_{i})\left\{ E_{t_{i+1}}[\text{log}(p(Y|\theta))]\right.\\ &\quad \left.+E_{t_{i}}[\text{log}(p(Y|\theta))] \right\}. \end{aligned} $$

The expectation with respect to the posterior at each temperature on the ladder can be approximated using the Monte Carlo estimate. For all the models we used a temperature schedule with *N*=10 according to an exponential ladder $t_{i} = \left (\frac {i}{N}\right)^{5}, i = 1,..., N$ as suggested in [25].

### Model over-fitting

The process of model selection described above helps guard against choosing over-parameterized models by penalizing them implicitly for higher dimensionality. This ability of Bayes factors to prioritize simpler models over complex ones has also been discussed elsewhere [28, 41].

However, as we consider relative goodness of fit amongst models, there might still be an argument that the best chosen model does over-fit the data. One way to test model over-fitting is cross-validation [44]. In such an approach, usually, we can envisage excluding some of the data (validation set) during model fitting step and then testing the accuracy of the model on this held-out data set. An over-fit model would perform well on the fitted data but poorly on the held-out dataset.

However, as we deal with a spatially correlated dataset, cross-validation becomes more difficult as leaving out an observation does not remove all the associated information. In order to compute a cross-validation statistic, we use an iterative procedure. We use the mean log-likelihood as a measure of prediction accuracy.

1. We fit the model to the data *y*_1_,⋯,*y*_*m*_, where *m* is chosen such that 1,⋯,*m* corresponds to the first 60% of the data, drawn sequentially across the embryo axis.
2. We use the fitted model to predict for the next 5% of observations and compute the log-likelihood.
3. Repeat steps 1 & 2, adding 5% of the data set to training set and predict the next 5%.
4. Finally, compute the mean log-likelihood from the predictions made above.

As our data is stratified, we ensure that the training set draws evenly from expression observation of the gap genes, i.e. we pick the initial 60% of the observations from each of the four gap genes to train the model. Similarly, predictions are made on the next 5% of the observations for each gap gene.

The models, solver and MCMC sampler were coded using the python programming language. PyMC [45] was used for certain diagnostic visualizations. The code for reproducing the analysis is available on GitHub at the repository: https://github.com/asifzubair/BayesianModelSelection.

## Results

The *Drosophila* gap gene network has been the subject of intense study from both experimentalists and computational modelers. Despite this, efforts to compare proposed network hypothesis in a statistically rigorous manner have been few and far between. Here, we propose to use the Bayesian framework for doing parameter inference and model selection. The Bayesian framework permits one to do a fully probabilistic analysis of model system allowing one to account for uncertainty in parameter estimates and model fit. We employ an MCMC approach using the parallel tempering (PT-MCMC) sampler to do Bayesian analysis. This sampler not only allows for better convergence but also helps one to compute the thermodynamic estimator for marginal likelihood. Other sampling approaches for accelerating convergence like adaptive MCMC [46] and Hamiltonian Monte Carlo (HMC) [47] exist. However, these samplers require all the parameters to be continuous whereas the PT-MCMC sampler does not have such a restriction. In addition, they do not have the benefit of providing a natural way to estimate the marginal likelihood like the PT-MCMC sampler does. Using estimates of the marginal likelihood, we use Bayes factor to compare between models.

Papatsenko & Levine argued that if the gene expression model is robust to the parameter values, then a single set of robust parameters should provide good model fits. In keeping with this, we set parameters related to maximal synthesis (*α*), decay (*β*), cooperativity rates (*C*_*o*_) and diffusion (*D*) to be the same for all gap genes. In addition, we set the number of binding sites (*N*_*s*_) to be the same. This forms the base model of 6 parameters (Model A6). Thereafter, we introduce node specific parameters to account for unequal mutual repression between Hb-Kni (*K*_1_) and Gt-Kr (*K*_2_). This is Model B7. We further test the possibility of the node-specific parameter (*K*_3_) controlling Bicoid activation of three gap genes - Knirps, Hunchback and Giant. This is Model C8. In addition to this, certain studies have indicated the possibility of Bicoid activating Kruppel [48, 49], we also test for the evidence of this by adding an extra edge to Models B7 and C8. These are models D7 and D8. All model specifications are described in Table 1.

**Table 1:** Specifications for all 6 models evaluated

Models	A6	B7	B7r	C8	D7	D8
Global parameters:						
Affinity(logKa)	*K*	*K*	*K*	*K*	*K*	*K*
Cooperativity	*C*_*o*_	*C*_*o*_	*C*_*o*_	*C*_*o*_	*C*_*o*_	*C*_*o*_
Binding Sites	*N*_*s*_	*N*_*s*_	*N*_*s*_	*N*_*s*_	*N*_*s*_	*N*_*s*_
Syn./Decay	*α*	*α*	*α*	*α*	*α*	*α*
Diffusion	D	D	D	D	D	D
Max. conc	50	50	50	50	50	50
Node-specific binding affinities:						
*Bcd* ^*A*^	*K*	*K*	*K*_3_	*K*_3_	*K*	*K*_3_
*Bcd* ^*R*^	*K*	*K*	*K*	*K*	*K*	*K*
*Cad* ^*A*^	*K*	*K*	*K*	*K*	*K*	*K*
*Hb* ^*A*^	*K*	*K*	*K*	*K*	*K*	*K*
*Hb* ^*D*^	*K*	*K*	*K*	*K*	*K*	*K*
*Hb* ^*R*^	*K*_1_	*K*_1_	*K*_1_	*K*_1_	*K*_1_	*K*_1_
*Gt* ^*R*^	*K*	*K*	*K*	*K*	*K*	*K*
*Kr* ^*R*^	*K*_1_	*K*_2_	*K*_1_	*K*_2_	*K*_2_	*K*_2_
*Kni* ^*R*^	*K*	*K*	*K*	*K*	*K*	*K*
*Tll* ^*R*^	*K*	*K*	*K*	*K*	*K*	*K*
Open Parameters:	6	7	7	8	7	8

Models D7 and D8 have an extra edge for the activation of Bicoid by Kruppel. Also shown is the break up of global and node-specific parameters for different models. *Hb*^*D*^ indicates parameter for the dual regulatory action of Hunchback on Kruppel

In their paper, Papatsenko & Levine [26] fit each of the models (A6, B7 and C8) separately by maximizing an objective function based on the correlation measured between the model and the data. They use the final correlation value to distinguish between the models. Their formulation and analysis showed that the gap gene network can be modeled using a more modular approach, involving two relatively independent network domains. In addition, they show close agreement of parameter estimates and experimentally observed values for most parameters. However, their approach to compare the models themselves is slightly problematic as it does not apply appropriate penalties for increasing model dimensionality. Bayes factors apply this penalty implicitly and so adhere to the notion of Occam’s razor of favoring simple hypothesis over complex ones. Moreover, Papatsenko & Levine do not offer a measure of statistical significance to justify model choice and rely on an ad-hoc notion of over-fitting. We enhance their fundamentally sound approach by allowing for statistically rigorous model selection and also allow for comparing competing network hypothesis.

### Efficient model solver

The approach of Papatsenko & Levine for solving the system of partial differential equations was to use a forward Euler integration loop in which diffusion is simulated by a Gaussian filter. However, the implementation of the solver was much too slow for a Bayesian analysis, where one may have to run upwards of a million iterations. To overcome this, we solved the system by the method of semi-groups. This gives rise to an iterative solution that can easily be vectorized and is numerically efficient. Our solver is an order of magnitude faster than the solver due to Papatsenko & Levine (Additional file 1, Fig. 2).

**Fig. 2:**
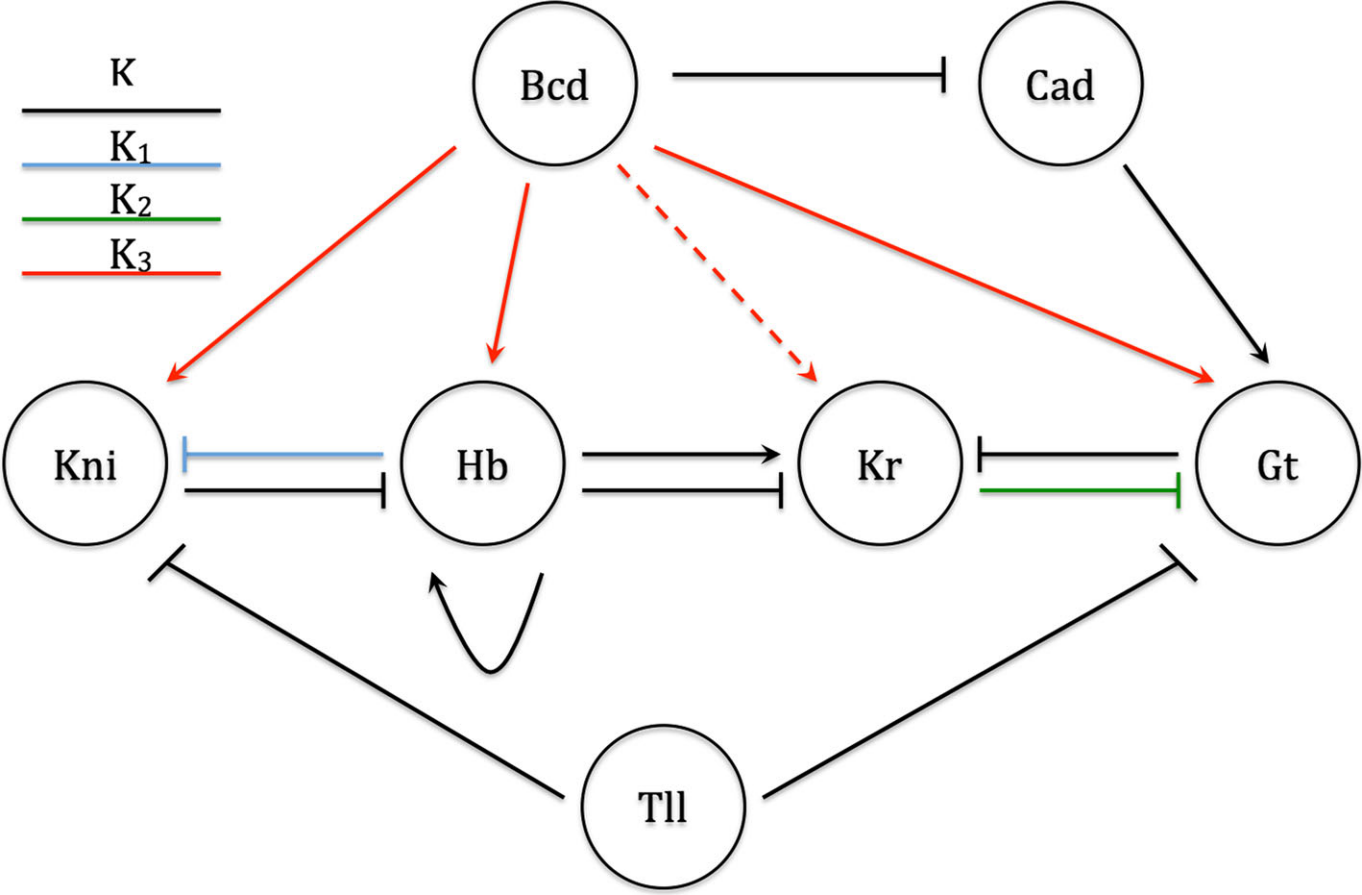
Gap gene network. Gap gene network showing regulatory interactions between maternal genes, Bicoid (Bcd) & Caudal (Cad), and gap genes (Knirps (Kni), Hunchback (Hb), Kruppel (Kr), Giant (Gt)). Two types of binding affinity parameters are shown - global (K) and edge-specific (*K*_1_,*K*_2_,*K*_3_). We also investigate evidence for Bicoid activation of Kruppel (shown as dashed arrow)

### Convergence of MCMC runs

Time to convergence for MCMC samplers can be sensitive to initial start points. To overcome this, some approaches try to initialize the sampler from the MLE estimate of the likelihood function. This approach suffers from the same pitfalls as optimization algorithms, in that the sampler may not sample the whole likelihood space and the evidence of convergence may be misleading.

To ensure that the sampler had indeed converged, we initialized the chain from random start points drawn from a uniform prior. We used the Gelman-Rubin statistic [50] to monitor convergence of the chains. This diagnostic uses multiple chains to check for lack of convergence, and is based on the notion that if multiple chains have converged, by definition they should appear very similar to one another. The Gelman-Rubin statistic uses an analysis of variance approach to assessing convergence by calculating both the between-chain variance and within-chain variance to assess whether chains have indeed converged. We used the gelman.plot() function from the R [51] package coda [52] to plot the Gelman-Rubin statistic. It calculates the Gelman-Rubin shrink factor (*R*) repeatedly, first calculating with 50 observations and then adding bins of 10 observations iteratively. For convergence, we would ideally want the shrink factor to be below 1.2.

**Fig. 3:**
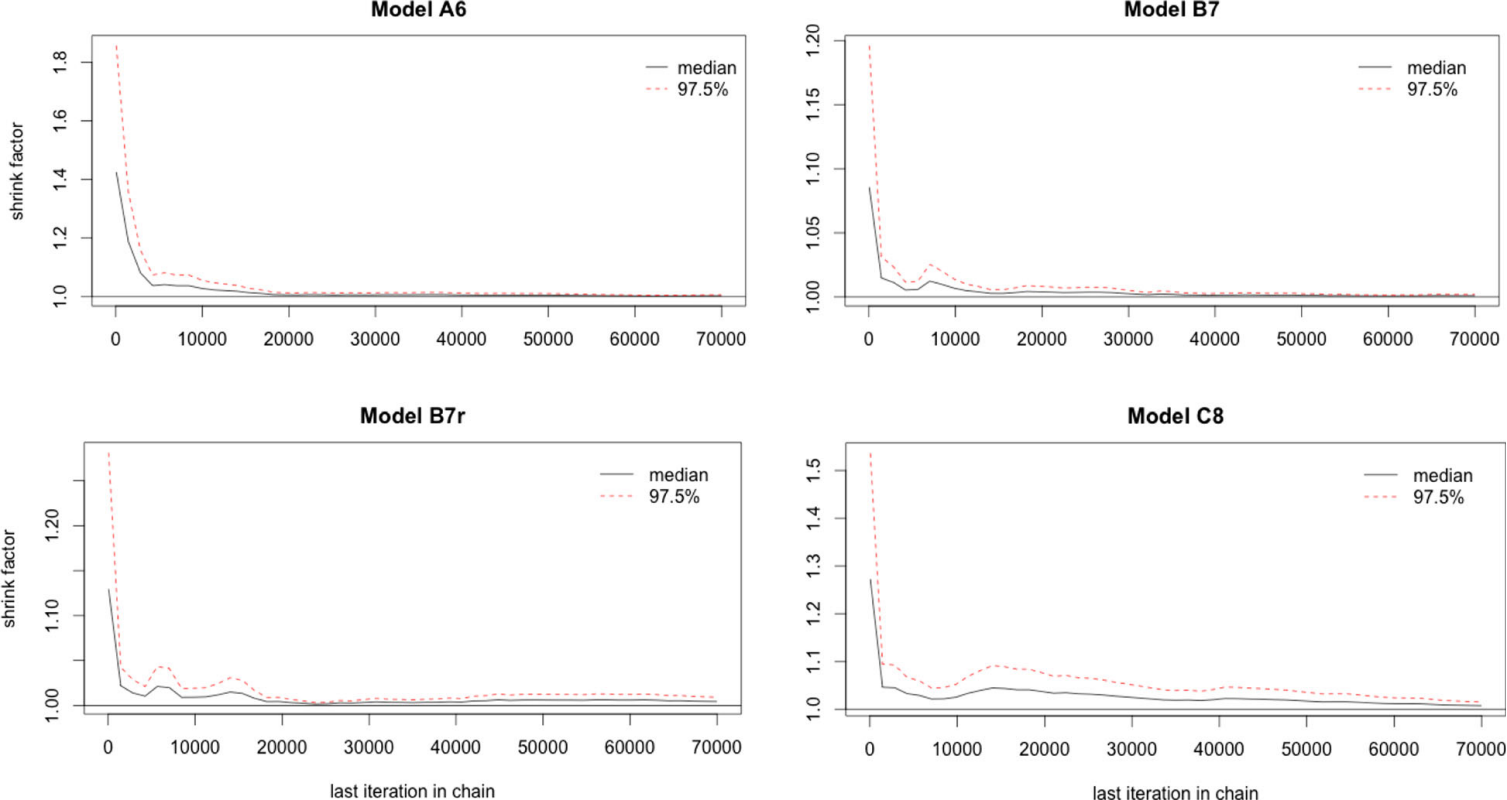
MCMC convergence diagnostics. Gelman plot showing the evolution of the gelmna-rubin statistic for four models (A6, B7, B7r, C8) as a function of iterations. The diagnostic metric was evaluated for 10 independent chains with random start points for each model. Values less 1.2 imply good mixing of the chains. Diagnostic plots for other models can be found in Additional file 1

Posteriors samples generated by fitting the data to simulated data showed evidence of confounding between a set of parameters (Additional file 1, Fig. 3). So, we used the convergence criteria on the likelihoods of the models. Figure 3 shows the Gelman-Rubin statistic for four models. We see that the shrink factor drops sharply with number of iterations of the chain for all models. This implies that the chains have, indeed, converged.

### Marginal likelihood and Bayes factors

**Fig. 4:**
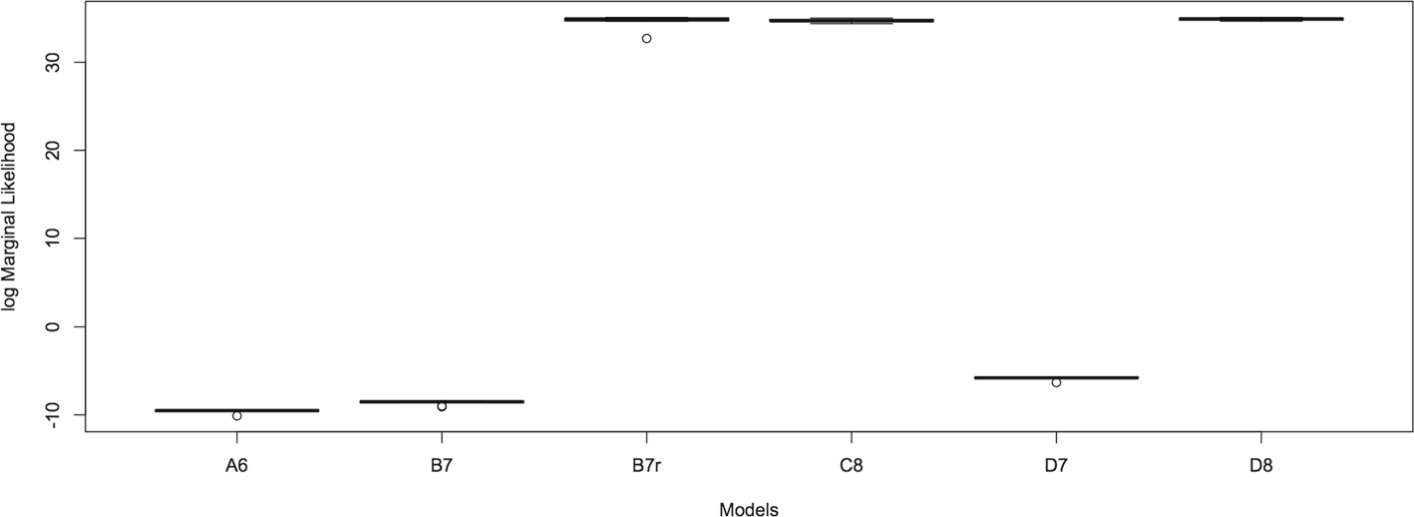
Log marginal likelihood estimates. Thermodynamic estimate of the logarithm marginal likelihood for all models. Estimates were generated for 10 independent runs for each model and show low variance. Difference between the estimates for models reveals the log Bayes factor that can be used for model comparison (see Table 2). We see that addition of a node specific-parameter for Bicoid improves the model fit in a statistically significant manner

The output from the PT-MCMC at different temperatures was used for computing the marginal likelihood. For each model, we computed the estimate of the log of the marginal likelihood estimate from 10 parallel runs using thermodynamic integration (see methods). 10 independent runs of the sampler were used to compute the estimate and are shown in Fig. 4. The estimates show low variability. Based on the log of the marginal likelihood, it is straightforward to compute the Bayes factors (see Table 2 for interpretation of Bayes factors). We find that the Bayes factor for model C8 over model B7 is very strong. However, there isn’t strong evidence supporting model D8 over model C8. This leads us to believe that there isn’t strong evidence from the data to support Bicoid activation of Kruppel. However, the data does support a different distribution for the node specific parameter describing the binding affinity of Bicoid. This is evidenced by the fact that there isn’t strong evidence for model C8 over model B7r.

**Table 2:** Criteria due to Kass & Rafferty [22] for interpretation of Bayes factor as evidence support categories

2*log*_*e*_(*B*)	*B*	Evidence against *H*_0_
0 to 2	1 to 3	Not worth more than a bare mention
2 to 6	3 to 20	Substantial
6 to 10	20 to 150	Strong
>10	>150	Very strong

### Gene expression profiles

**Fig. 5:**
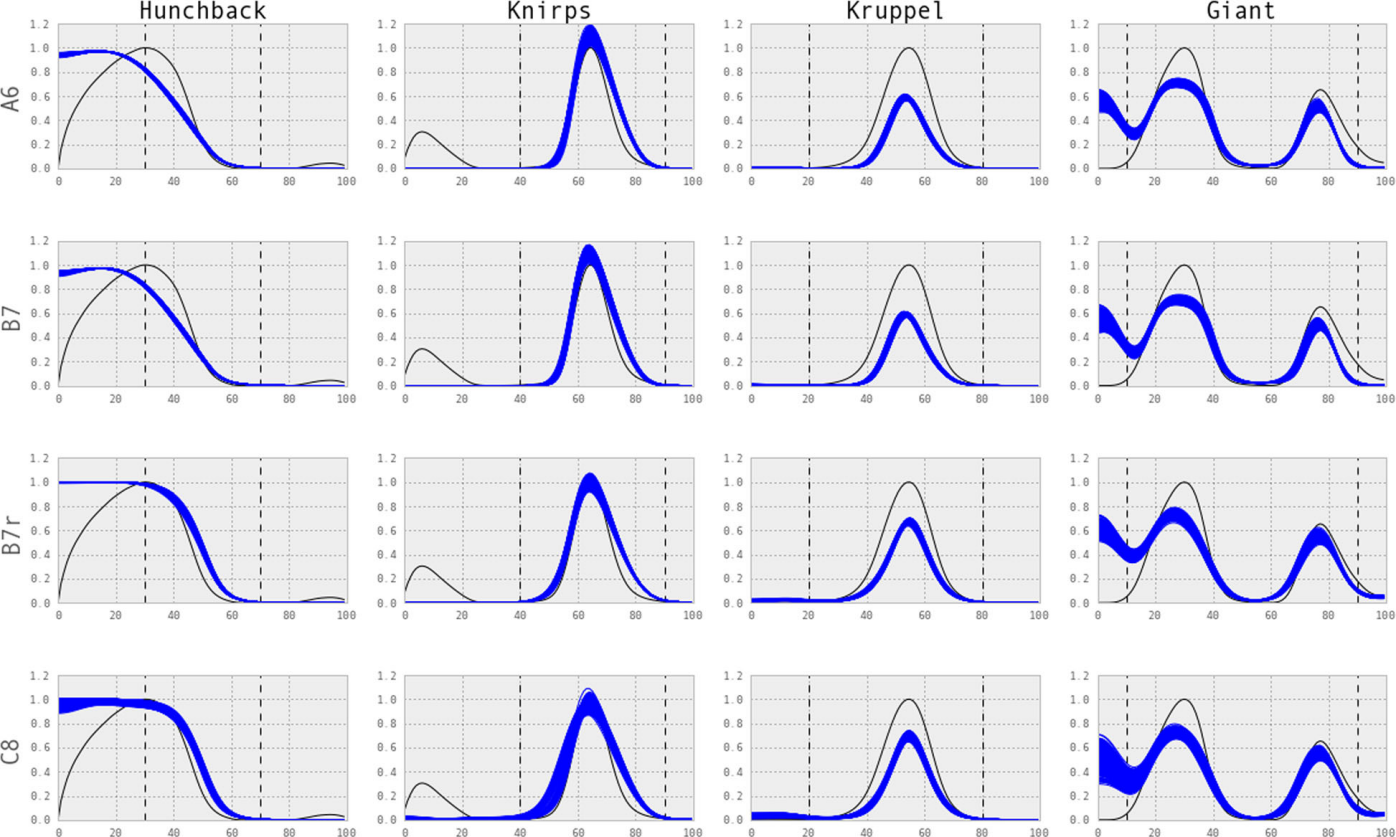
Gene expression profiles. Gene expression profiles for Models A6, B7, B7r, C8. Black lines show observed values and blue lines are model outcomes by sampling parameters from the joint posterior. For each model, 100 samples were drawn from the joint posterior of model parameters. Vertical dotted lines show domains over which the likelihood was computed

Model outcomes were generated by sampling from the joint posterior of the model parameters. For each model, 100 samples were taken from the joint distribution and the model outcomes generated by using the parameter set (see Fig. 5). The basic model with 6 parameters (model A6) also captures the main features of the expression pattern, showing that the inference procedure is able to sample from the correct posterior. As the likelihood is computed only within certain domains (shown by vertical dotted lines for each gap gene in Fig. 5), model outcomes show higher variability outside these domains. Most noticeable is the posterior shift of Hunchback expression seen in models B7r and C8. This shows that a different distribution of Bicoid binding affinity from the global affinity parameter is sufficient to capture the characteristic expression curve of Hunchback. Increasing the number of parameters from 7 to 8 improves the model fit (as judged from the marginal likelihood), it does so not in a statistically significant manner. The model outcomes for models D7 & D8, that describe models with an extra regulatory edge for Bicoid, can be found in Additional file 1.

### Over-fitting analysis

We tested the best performing model (according to Bayes factor criteria), model B7r, for over-fitting. We used a modified cross-validation (CV) approach for testing over-fitting (see methods). In each CV-fold, we fit the model to the training set and then draw 100 samples from the posterior parameter distribution. The posterior samples are used to predict values for the held out set. We use the mean log-likelihood metric as prediction accuracy measure. As a Gaussian error model is used, the mean log-likelihood is proportional to the residual error in this case. The mean log-likelihood for the cross validation set is 0.314 (±0.024). The mean log-likelihood using samples from posterior parameter distribution generated using the complete data is 0.326 (±0.061). Using a Student’s t-test with Welch modification, we found the difference in means to not be statistically significant (*P*>0.05) indicating that the model doesn’t over-fit the data.

## Discussion

Recovering gene regulatory network information from expression data is a key problem in systems biology. Particularly in the study of the segmentation pathway for early *Drosophila* embryo, various modeling approaches have been taken [12, 17, 26]. However, most of these modeling approaches rely on the assessment of a single candidate model. This sort of approach has been previously argued against [53] as it doesn’t pay heed to competing hypotheses and hence, other plausible explanations. In addition, inference in these approaches rely on optimization techniques which do not account for uncertainty in experimental measurements. Optimization approaches try to offer measures of parameter certainty through sensitivity analysis but, barring certain studies [54], the issue of comparing models has been largely unadressed.

We do note that there have been some attempts [55] at doing model selection in the *Drosophila* embryo. However, the application of a structured framework in which models can be compared is still elusive. Doing the analysis in a Bayesian framework provides a more standard procedure to address both the issues of performing inference regarding different models and to assess the certainty of parameter estimates. An important issue when working with dynamical models is the issue of identifiability [49, 56–58] - the ability to uniquely estimate parameters of the model given the data. In the Bayesian context, a priori identifiability issues can be detected by examining the covariance structure of the full parameter posterior distribution. Parameters that are confounded will be tightly correlated. Identifiability issues can be surmounted by providing a more informative prior that more tightly constrains confounded parameters. In our case, however, we have chosen to work with uniform priors to indicate that our knowledge of the system is still evolving. Indeterminacy of model parameters are incorporated into the marginal likelihood, allowing one to still perform model selection. However, parameter relationships can still uncover important mechanisms. In our study, we find that the parameters for binding affinity and number of sites are negatively correlated (Additional file 1: Figure S3). Such a relationship is expected as it indicates that a transcription factor can modulate gene expression by either binding strongly to a few sites or through weak binding to multiple sites. Similar to our study, Chertkova et al. [59], show that loss of transcription factor binding sites in in silico models results in increase in binding affinity of transcription factors, supporting negative correlation between these parameters in order to maintain gene expression.

In the Bayesian framework, Bayes factors provide a means of doing model selection and have been employed to compare between ODE based models [25, 42, 60, 61]. We show here that similar approaches can be used for doing model selection in the context of PDE models for spatial patterning. An advantage of the Bayesian model selection paradigm using Bayes factors is that it doesn’t require models to be nested, i.e models need not follow a set hierarchy where all models may be derived from an extended parameterized model. This particularly advantageous when we attempt to test hypotheses involving different network topologies. Samples from the posterior of parameter distribution were generated using the parallel tempering (PT-MCMC) sampler. This sampling approach can be easily combined with the numerically stable thermodynamic integration method to estimate marginal likelihood for each of the competing models. These estimates in turn can be used to compute Bayes factors. Our analysis shows that besides the global binding affinity parameter, a different node-specific parameter is required for describing the regulatory effect of Bicoid on its target genes. This may point to the fact that the molecular mechanism of activation by Bicoid is different from other maternal/gap genes. The node-specific Bicoid binding affinity parameter helps account for a posterior shift of Hunchback expression. A candidate hypothesis for the activation of Kruppel by Bicoid was also tested for. Our analysis offers little support for the activation of Kruppel by Bicoid.

We point out that as the computation of posterior probabilities in Bayesian analysis involves integration over high-dimensional parameter spaces, sampling from higher dimensions becomes increasingly difficult. This is a particular limitation for the large parameter models that we see in systems biology. While there has been some progress in Bayesian parameter estimation in high-dimensions [62], this problem is far from solved. However, there might be some justification in criticism that these high-dimension models also tend to be over-parameterized and thus too flexible. One approach would be do a hierarchical Bayesian analysis [63] to constrain parameter sets in order to prevent the problem of over-fitting and estimation in higher dimensions.

## Conclusion

This study aims to elaborate on a Bayesian framework for conducting model selection in context of the *Drosophila* early developmental segmentation pathway. In particular, we focus on identifying regulatory interactions for the gap gene network. Our study seeks to provide a statistical framework in which predicted experimental hypothesis can be tested. In addition, the model selection procedure also ensures that a minimal model for gap gene expression can be formulated. In order to conduct such an analysis, we provide an efficient solver for the Papatsenko-Levine formulation. In conclusion, we find that a seven parameter model with a node-specific binding affinity to describe regulatory action of Bicoid on the gap genes explains the data adequately.

## Additional file

Additional file 1 Supplementary material describing Papatsenko-Levine formalism with description of regulatory framework, full derivation of the solver for the general form of the model, runtime comparison between solvers, model fit to simulated data, convergence properties and output for models D7 & D8. (PDF 728 kb)

## Abbreviations

AP: Anterior-Posterior
Bcd: Bicoid
Gt: Giant
Hb: Hunchback
HMC: Hamiltonian monte carlo
Kni: Knirps
Kr: Kruppel
MCMC: Markov Chain monte carlo
MH,: Metropolis-hastings
MLE: Maximum likelihood estimate
ODEs: Ordinary differential equations
PDEs: Partial differential equations
PT-MCMC: Parallel tempering markov chain monte carlo
Tll: Tailless
