# Supplementary material for: Bayesian model selection for the Drosophila gap gene network
Source: BMC Bioinformatics. 2019 Jun 13;20:327. doi: 10.1186/s12859-019-2888-0 (PMC6567646; doi:10.1186/s12859-019-2888-0)
Supplement: Supplementary file 1 — Supplementary material describing Papatsenko-Levine formalism with description of regulatory framework, full derivation of the solver for the general form of the model, runtime comparison between solvers, model fit to simulated data, convergence properties and output for models D7 & D8. (PDF 728 kb) [file 12859_2019_2888_MOESM1_ESM.pdf]

Supplementary Material
Bayesian Model Selection for the *Drosophila* Gap Gene Network

Asif Zubair, I. Gary Rosen, Sergey V. Nuzhdin, Paul Marjoram

Models

Regulatory Framework

For a group of N_s cooperating (C_o - cooperativity fold, $C_o \epsilon [1, \infty]$) equal binding sites, all with binding constant K, the probability of occupancy of at least one site in the group is equal to [1]:

$$p([A], K, C_o, N_s) = 1 - \frac{C_o}{C_o + (1 + C_o K[A])^{N_s} - 1}.$$

According to this framework, p is proportional to the probability of activation of a gene, regulated by the transcriptional activator A. If A is a transcriptional repressor, the the probability of repression of the downstream gene is $1-p$. If gene expression is outcome of several regulatory events and they are all required for expression, then the synthesis rate, P, of the gene is given by the product of activation from i site arrays for i activators and repression from j site arrays for j repressors as follows [2]:

$$P = \prod_i p_i^{act} \prod_j \left(1 - p_j^{rep}\right),$$

where p_i^{act} is the occupancy probability of activator i and p_j^{rep} is the occupancy probability of activator j. Input integration using multiple independent activators can be expressed using the following:

$$P = 1 - \prod_i \left(1 - p_i^{act}\right).$$

Here, we give an example to mathematically construct the regulatory information for the gap gene Giant (Gt). For more details, please refer to [3].

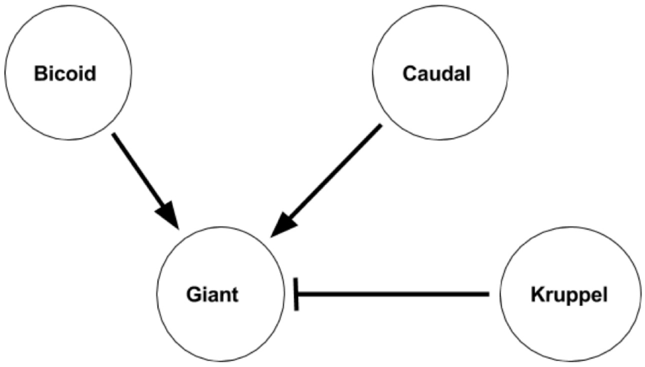

Figure 1: Regulatory interactions of Giant. Giant is activated by the maternal genes, bicoid and caudal, in a OR manner. It is repressed by the gap gene, Kruppel.

As depicted in fig. 1, Giant (Gt) is activated by the presence of either of the maternal genes, Bicoid or Caudal, (OR activation). In addition, Giant is repressed by the action of of Kruppel.

Based on this scheme, we can formulate the rate of Giant production as:

$$P^{Gt} = (1 - (1 - p^{Bcd})(1 - p^{Cad}))(1 - p^{Kr}),$$

$$P^{Gt} = (1 - \frac{C_o^{Bcd}}{C_o^{Bcd} + (1 + C_o^{Bcd}K^{Bcd}[Bcd])^{N_s^{Bcd}} - 1} * \frac{C_o^{Cad}}{C_o^{Cad} + (1 + C_o^{Cad}K^{Cad}[Cad])^{N_s^{Cad}} - 1})$$
$$* (\frac{C_o^{Kr}}{C_o^{Kr} + (1 + C_o^{Kr}K^{Kr}[Kr])^{N_s^{Kr}} - 1}).$$

Model Equations

Assuming the constants defined in Table 1 of the paper, we provide the complete partial differential equations associated with each of the gap genes.
Hunchback (Hb):

$$\frac{\partial}{\partial x}[Hb] = \alpha \left((1 - \frac{C_o}{C_o + (1 + C_o K_3[Bcd])^{N_s} - 1})(1 - \frac{C_o}{C_o + (1 + C_o K[Hb])^{N_s} - 1})(\frac{C_o}{C_o + (1 + C_o K[Kni])^{N_s} - 1}) \right)$$
$$- \beta[Hb] + D/L^2 \frac{\partial^2 [Hb]}{\partial x^2}.$$

Knirps (Kni):

$$\frac{\partial}{\partial x}[Kni] = \alpha \left((1 - \frac{C_o}{C_o + (1 + C_o K_3[Bcd])^{N_s} - 1})(\frac{C_o}{C_o + (1 + C_o K_1[Hb])^{N_s} - 1})(\frac{C_o}{C_o + (1 + C_o K[Tll])^{N_s} - 1}) \right)$$
$$- \beta[Kni] + D/L^2 \frac{\partial^2 [Kni]}{\partial x^2}.$$

Kruppel (Kr):

$$\frac{\partial}{\partial x}[Kr] = \alpha \left((1 - \frac{C_o}{C_o + (1 + C_o K[Hb])^{N_s} - 1})(\frac{C_o}{C_o + (1 + C_o K[Hb])^{N_s} - 1})(\frac{C_o}{C_o + (1 + C_o K[Gt])^{N_s} - 1}) \right)$$
$$- \beta[Kr] + D/L^2 \frac{\partial^2 [Kr]}{\partial x^2}.$$

Giant (Gt):

$$\frac{\partial}{\partial x}[Gt] = \alpha \Bigg((1 - (1 - \frac{C_o}{C_o + (1 + C_o K_3[Bcd])^{N_s} - 1} * \frac{C_o}{C_o + (1 + C_o K[Hb])^{N_s} - 1}))$$
$$(\frac{C_o}{C_o + (1 + C_o K_2[Kr])^{N_s} - 1})(\frac{C_o}{C_o + (1 + C_o K[Tll])^{N_s} - 1}) \Bigg) - \beta[Gt] + D/L^2 \frac{\partial^2 [Gt]}{\partial x^2}.$$

To produce the base model A6, K_3 is set to equal K and K_2 is set to equal K_1. Further models are derived according to specifications in Table 1 of the main section. To include the activation of kruppel by bicoid, the equation of kruppel above is modified to the following:

$$\frac{\partial}{\partial x}[Kr] = \alpha \Bigg((1 - \frac{C_o}{C_o + (1 + C_o K_3[Bcd])^{N_s} - 1})(1 - \frac{C_o}{C_o + (1 + C_o K[Hb])^{N_s} - 1})$$
$$(\frac{C_o}{C_o + (1 + C_o K[Hb])^{N_s} - 1})(\frac{C_o}{C_o + (1 + C_o K[Gt])^{N_s} - 1}) \Bigg) - \beta[Kr] + D/L^2 \frac{\partial^2 [Kr]}{\partial x^2}.$$

Here, $[Bcd], [Tll]$ are the concentrations of maternal genes bicoid and tailless respectively. All concentrations are function of space and time, i.e.:

$$[A] \equiv [A](x, t)$$

Normalization

We assume equal synthesis rates for all four gab genes. This value is also same as the deacy rate for each of the gap genes. Further, Papatsenko and Levine [3] assumed that the gap genes

undergo similar activation at the beginning of their expression. This assumption can be handled by applying a normalization constant to the production term. Specifically, we pre-multiply the synthesis rate of gap gene $[A]$ with ω^A:

$$\frac{\partial}{\partial t}[A] = \omega^A \alpha P_A^{act}(1 - P_A^{rep}) - \beta[A] + D/L^2 \frac{\partial^2[A]}{\partial x^2},$$
$$\omega^A = \frac{1}{Max(P^{act}(x|t=0, K, C_o, N_s))}.$$

Model Solution

We provide here the complete solution to the reaction-diffusion equation describing gap gene expression. We first do a change of variables to dimensionless space $x \to x/L$, where L is the length of the embryo, and rewrite the equation as:

$$\begin{aligned}\frac{\partial}{\partial t}u_i(x,t) &= \alpha P_i^A(1 - P_i^B) - \beta u_i(x,t) + D/L^2 \frac{\partial^2 u_i(x,t)}{\partial x^2}, \\ i &= Hb, Kr, Kni, Gt, \\ u'(0,t) &= u'(1,t) = 0, u' = \frac{\partial u}{\partial x}, \\ 0 &< x < 1, 0 < t < T.\end{aligned} \tag{1}$$

Stated this way, the expression of gap genes is a system of non-linear partial differential equations (PDEs). For convenience, in the following derivation we drop the subscript i and set $f = \alpha P_i^A(1 - P_i^B)$ to capture the non-linearity in the system. The time differential of the concentration, u, is represented by u_t. Thus, we can write the above equation in the weak form as:

$$< u_t, v > \; = \; < f, v > -\beta < u, v > +D/L^2 < u_{xx}, v > .$$

Integrating by parts, we can re-write the above as:

$$< u_t, v > \; = \; < f, v > -\beta < u, v > -D/L^2 < u_x, v_x > .$$

We use the finite element subspace, $V_h = span\{\phi_i | 1 \leq i \leq n\}$ and approximate the solution with $u^n = \sum_{j=0}^{n} u_j \phi_j$ where, for $i = 1 \cdots n-1$:

$$\phi_i(x) = \begin{cases} nx - (i-1), & \frac{i-1}{n} \leq x \leq \frac{i}{n} \\ (i+1) - nx, & \frac{i}{n} \leq x \leq \frac{i+1}{n} \\ 0, & \text{otherwise} \end{cases}$$

and

$$\phi_0(x) = \begin{cases} 1 - nx, & 0 \leq x \leq \frac{1}{n} \\ 0, & \text{otherwise,} \end{cases}$$

$$\phi_n(x) = \begin{cases} nx - (n-1), & \frac{n-1}{n} \leq x \leq 1 \\ 0, & \text{otherwise.} \end{cases}$$

Given the basis, we can write the finite element method as:

$$\sum_{j=0}^{n} (u_j)_t < \phi_j, \phi_i > = < f, \phi_i > - \sum_{j=0}^{n} u_j(\beta < \phi_j, \phi_i > +D/L^2 < \phi_j', \phi_i' >).$$

Rewriting in matrix form, where the superscript, n , now indicates that we are working in the subspace V_h,

$$\begin{aligned} M^n u_t^n &= -(\beta M^n + (D/L^2)K^n)u^n + F \\ u_t^n &= -(\beta I + (D/L^2)(M^n)^{-1}K^n)u^n + (M^n)^{-1}F \\ u_t^n &= -A^n u^n + F^n. \end{aligned} \tag{2}$$

Here, $F = (< f, \phi_1 >, < f, \phi_2 >, \cdots, < f, \phi_n >)'$ and

$$\begin{aligned} M^n &= [M^n_{ij}] = [< \phi_j, \phi_i >], \\ K^n &= [K^n_{ij}] = [< \phi'_j, \phi'_i >], \\ A^n &= \beta I + (D/L^2)(M^n)^{-1}K^n \\ F^n &= (M^n)^{-1}F. \end{aligned}$$

We can now use the integrating factor method to solve the above ordinary differential equation in (2). Consider an interval τ in which we study the system. We can divide the total time T into T/τ such intervals and examine the system at the kth step where $u^n_k = u^n(k\tau)$. Using this formulation, we write:

$$\begin{aligned} u^n_{k+1} &= u^n((k+1)\tau) \\ &= e^{A^n\tau}u^n(k\tau) + \int_{k\tau}^{(k+1)\tau} e^{A^n((k+1)\tau - s)}F^n(u^n(s))ds \\ &\approx e^{A^n\tau}u^n(k\tau) + \int_{k\tau}^{(k+1)\tau} e^{A^n((k+1)\tau - s)}dsF^n(u^n(k\tau)) \\ &\approx e^{A^n\tau}u^n(k\tau) + \int_0^{\tau} e^{A^n s}dsF^n(u^n_k) \\ \Rightarrow u^n_{k+1} &\approx \Phi^n u^n_k + B^n F^n(u^n_k) \end{aligned} \tag{3}$$

As A^n is invertible, the integral $B^n = \int_0^\tau e^{A^n s}ds$ is evaluated to be $(A^n)^{-1}(e^{A^n\tau} - I)$. Equation (3) now provides an iterative solution for the PDE expressed in (1).

Runtime Comparison

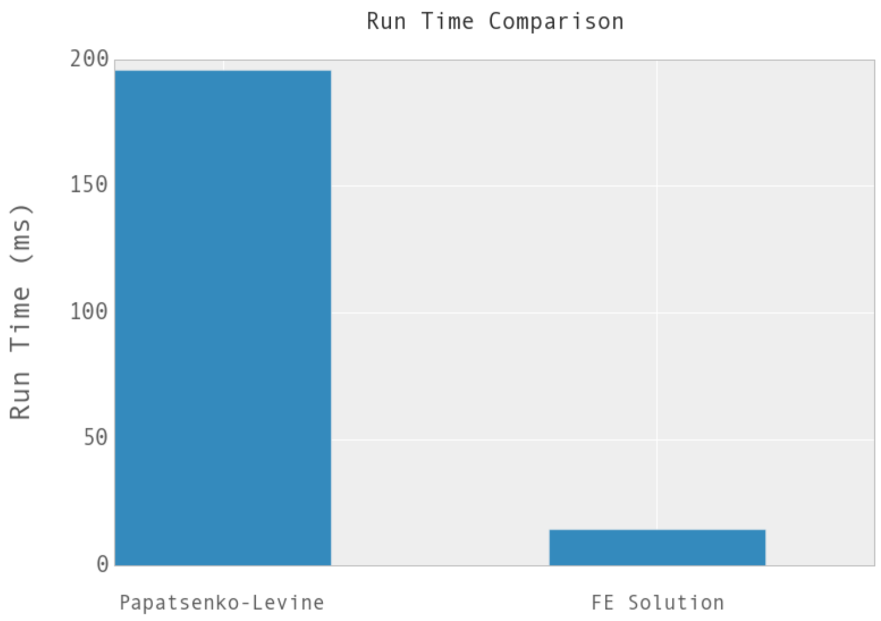

Figure 2: Runtime comparison of solver due to Papatsenko-Levine and using a finite element solution (our method). Our solver has a speed up of around 20X when computed over 10 function evaluations.

Fitting to simulated data & identifiability

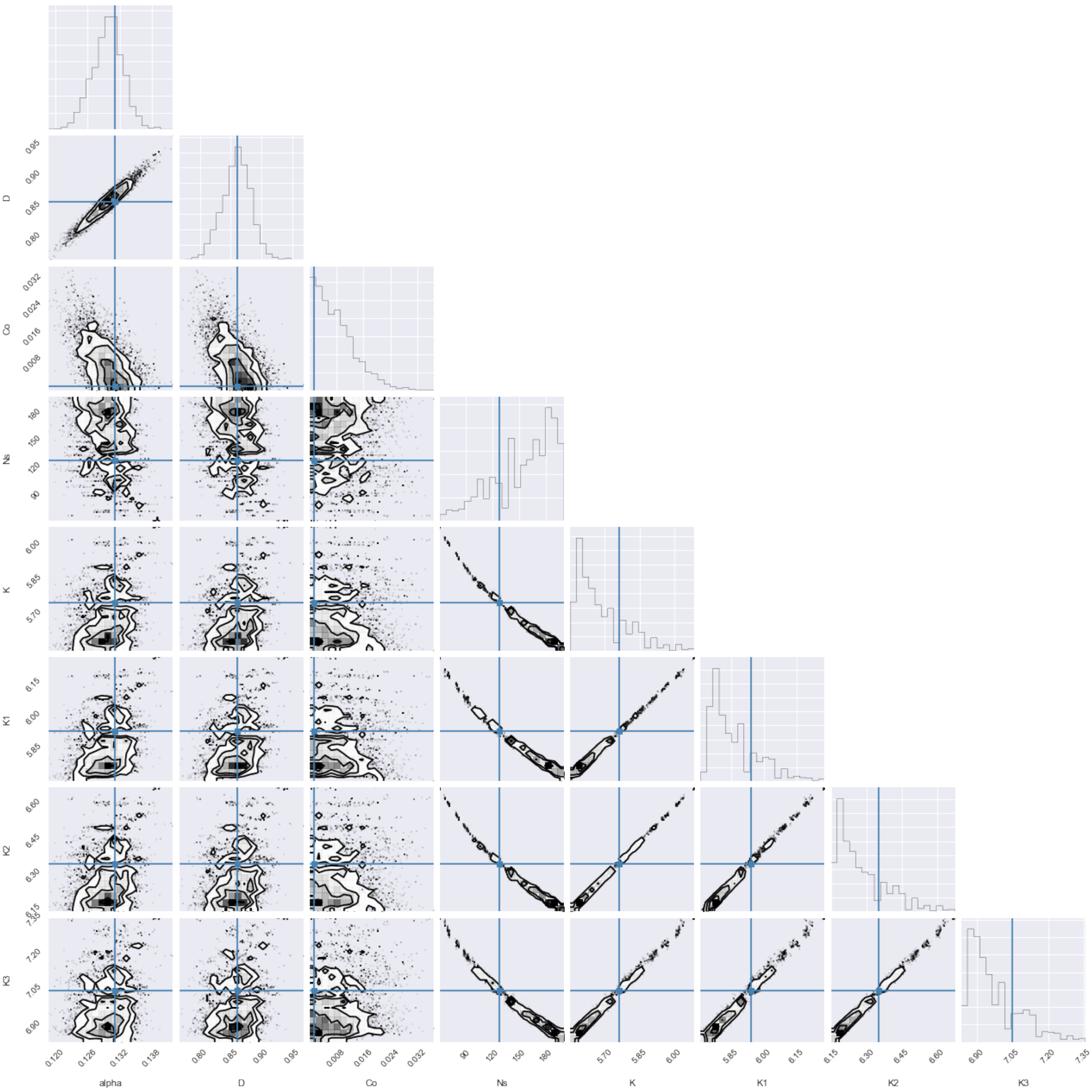

Figure 3: Corner plot [4] showing pair-wise joint densities of parameter for model C8. Simulated data, generated by adding noise to the output of the model with known parameters, was used to fit the model. The true values of the parameters are shown with blue lines. We see a strong negative correlation between the number of binding sites parameters, N_s, and the binding affinity parameters, K, K_1, K_2, K_3.

To investigate the structural properties of the Papatsenko-Levine formalism, we fit the model C8 (largest number of parameters) to simulated data generated with a known parameter set. The simulated data is contaminated with Gaussian noise having zero mean and 0.1 variance. The parallel tempering sampler was used to fit the model to simulated data using 100,000 generations. As can be seen in figure 3, the parameters α, D and Co are recovered and show no confounding. However, it can be seen that the parameter for number of sites parameters (Ns) is negatively

correlated with binding affinities ($K1$, $K2$, $K3$). The binding affinities themselves are positively correlated. This points towards structural identifiability issues, but also is slightly intuitive as weaker binding affinity can be compensated by a increase in the number of 'weak' binding sites.

We also use a non-parametric bootstrap-based algorithm [5][6] to test for structurally non-identifiable parameters. In order to do this, 5000 fits with random initial values drawn from the parameter space were computed. The best 5% fits based on χ^2 values were analyzed non-parametrically for relations between parameters (fig. 4) that would indicate structural identifiability. The parameters for synthesis rate, α, diffusion rate, D and cooperativity, C_o, are found to be strongly identifiable. The binding affinities K_1, K_2, K_3 show some correlation with the number of sites N_s parameter and are weakly identifiable. We also want to point out that any identifiability issues can be integrated out in the calculation of the marginal likelihood in a Bayesian framework and as such model selection can still be performed.

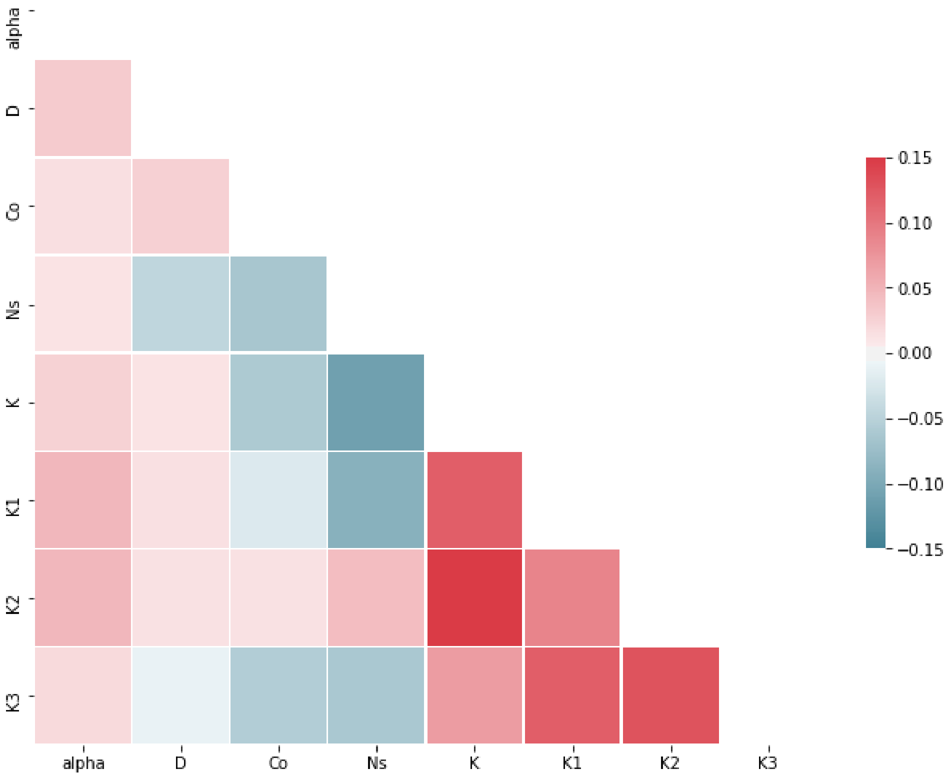

Figure 4: Identifiability: Non-parametric evaluation of parameter correlations based on sampling scheme that chooses the best 5% fits from random draws from whole parameter space. Parameters are weakly correlated indicating identifiability.

Diagnostic test & expression profile for models D7 & D8

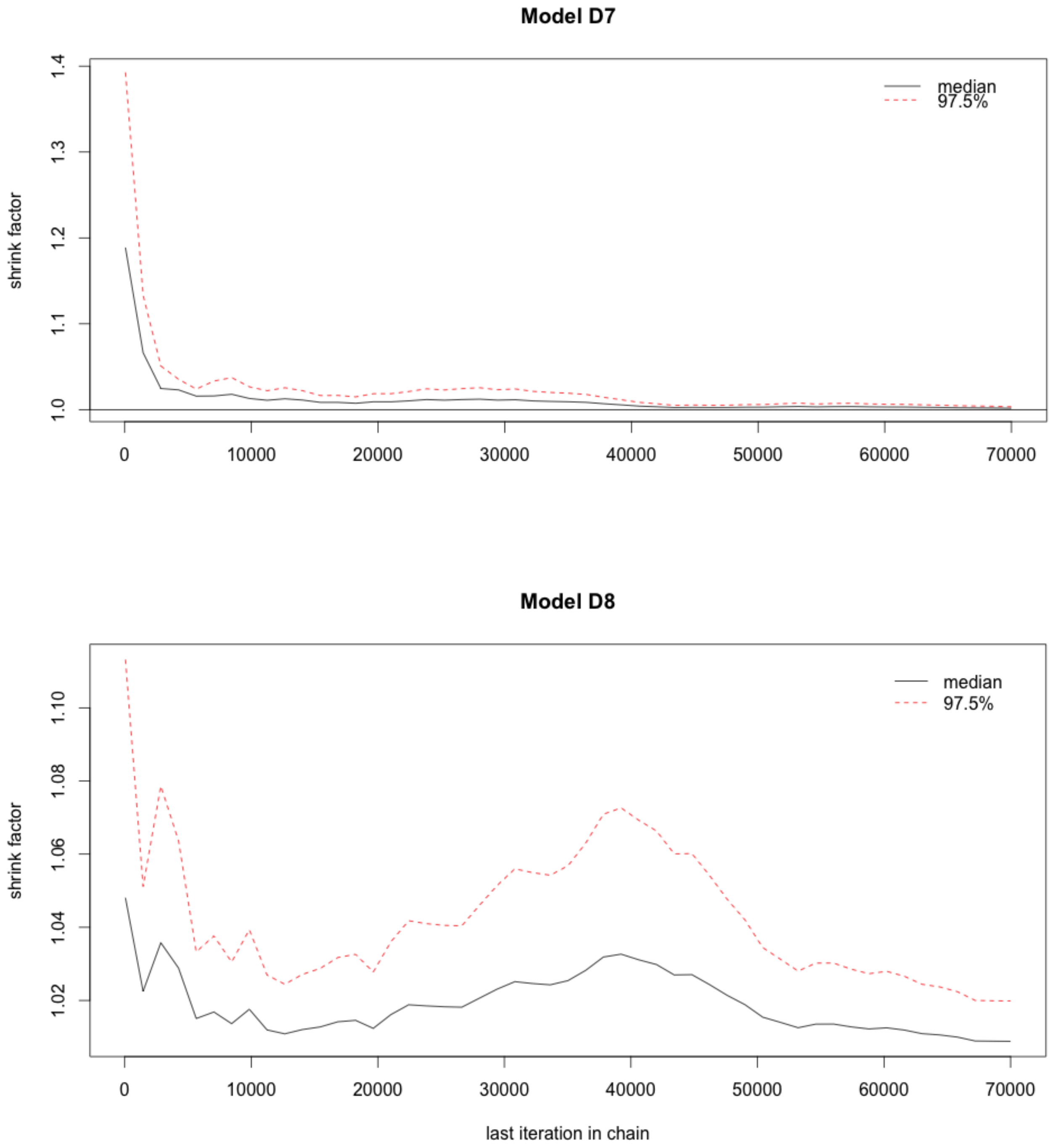

Figure 5: Gelman plot showing evolution of the diagnostic gelaman-rubin statistic for models, D7 and D8. Red dotted line is 97.5% confidence interval. 10 independent runs from random start points were used to test convergence. Median values below 1.2 signal convergence of the chains.

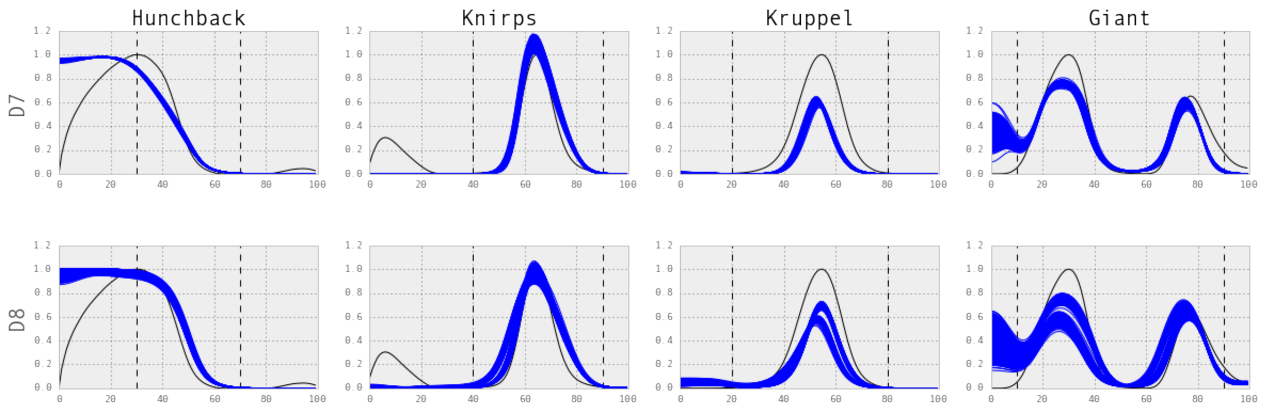

Figure 6: Gene Expression profiles for models D7, D8. Black lines show observed values and blue lines are model outcomes by sampling parameters from the joint posterior. For each model, 100 samples were drawn. Vertical dotted lines show domains over which the likelihood was computed.

References

[1] Zinzen, R.P., Senger, K., Levine, M., Papatsenko, D.: Computational models for neurogenic gene expression in the *Drosophila* embryo. Curr Biol **16**, 1358–1365 (2006)

[2] Bolouri, H.: Computational Modeling of Gene Regulatory Networks - A Primer. Imperial College Press, London (2008)

[3] Papatsenko, D., Levine, M.: The drosophila gap gene network is composed of two parallel toggle switches. PLoS One **6**(7), 21145 (2011)

[4] Foreman-Mackey, D.: corner.py: Scatterplot matrices in python. The Journal of Open Source Software **24** (2016). doi:10.21105/joss.00024

[5] Becker, V., Schilling, M., Bachmann, J., Baumann, U., Raue, A., Maiwald, T., Timmer, J., Klingmüller, U.: Covering a broad dynamic range: Information processing at the erythropoietin receptor. Science **328**(5984), 1404–1408 (2010). doi:10.1126/science.1184913

[6] Hengl, S., Kreutz, C., Timmer, J., Maiwald, T.: Data-based identifiability analysis of non-linear dynamical models. Bioinformatics **23**(19), 2612–2618 (2007). doi:10.1093/bioinformatics/btm382
